# Editorial: Advances and Applications of the EEG-fMRI Technique on Epilepsies

**DOI:** 10.3389/fneur.2021.827705

**Published:** 2022-01-13

**Authors:** Brunno Machado De Campos, Maria Centeno, Ana Carolina Coan, Fernando Cendes

**Affiliations:** ^1^Neuroimaging Laboratory, Department of Neurology, University of Campinas, Campinas, Brazil; ^2^Brazilian Institute of Neuroscience and Neurotechnology (BRAINN), São Paulo, Brazil; ^3^Department of Neurology, Hospital Clinic Barcelona, Barcelona, Spain

**Keywords:** editorial, EEG, fMRI, EEG-fMRI, connectivity, epilepsy

In this special issue of Frontiers in Neurology, we compiled a collection of articles focused on Applied Neuroimaging in Epilepsy. More specifically, this topic includes advances in the methodological development, application, and interpretation of the EEG-fMRI technique and its sub-fields. The main goal of this Research Topic was to collect data describing the progress of the technique's reliability on the localization of the epileptogenic zone. However, the articles herein also discuss scientific advances on EEG and fMRI data techniques individually, opening new horizons for data exploration and subsequent disease characterization. The use of EEG-fMRI for the localization of the epileptogenic zone relies on the understanding of each modality separately and the consequences of physical interactions intrinsic to this combination.

In this special issue, Bullock et al.'s systematic review summarizes the state of the art of EEG artifact removal proposing a standardized pipeline from hardware positioning to the most effective post-processing procedures for EEG data acquired during fMRI sessions. Complementary, the review article of Sadjadi et al. discusses the clinical applications of EEG-fMRI, elucidating its validity as a localization tool for the epileptogenic zone and the perspectives for the near future based on ongoing methodological advances.

In a study by Pinte et al., EEG electrode positioning relevant for EEG-source localization algorithms is explored. The MRI-compatible EEG-electrodes commonly used in EEG-fMRI sessions present minimal effect on MRIs. They propose an additional acquisition of an ultra-short echo-time image whereby the MR signal of the electrodes is detectable. This acquisition enables the localization of the electrodes through an automated pipeline based on a pre-trained deep-learning network, reaching good sensibility and accuracy. This approach has great potential for multimodal imaging, scalp EEG, and video-EEG analyses, as well as pre- and post-operative correlations with the surgical lacuna and EEG-fMRI data (1–3).

Piper et al. performed functional connectivity (FC) analyses of the anterior thalamic nucleus (ATN) using fMRI derived from EEG-fMRI acquisitions, demonstrating altered connectivity in children with refractory focal epilepsy using the EEG-informed interictal epileptiform discharges (IED) as a temporal regressor on the fMRI processing. The effect of IED events on brain function is a common bias in functional connectivity studies. Although IEDs are not associated with evident clinical manifestations, studies using PET and even EEG-fMRI could demonstrate that they alter basal hemodynamics (or rest condition), with the potential to bias analysis with the random prevalence of epileptiform events. Currently, the effects of IEDs are not considered in most FC studies (4). Accounting for IED effects improves the accuracy of connectivity studies and may offer some insight into the role of important therapeutic targets such as the ATN (5).

The FC based on fMRI acquired with EEG is also the subject of studies by Kassinopoulos et al., and Fu et al.. The first evaluates the relation between heart rate variability (HRV), a metric recently associated with refractory seizures and the severity of the epilepsies, and large-scale FC metrics. They describe relations between the HR and thalamic signals and connectivities, suggesting that these associations can play a role in the cardiac and blood pressure dysfunctions involved in SUDEP. Fu et al. explored FC in patients with refractory mesial temporal lobe epilepsy (MTLE) and benign epilepsy with centrotemporal spikes (BECT) compared to healthy controls. The findings showed different aberrations in the network-based interactions in MTLE and BECT, accessed throughout distinct global metrics, reinforcing that epileptic sub-syndromes should be considered distinct network diseases (6).

Chaudhary et al., Cohen et al., Ebrahimzadeh et al., Labounek et al., and Suarez et al. focused on the localization of the epileptogenic zone (EZ) and its related networks. Chaudhary et al. performed intracranial EEG during fMRI sessions (icEEG-fMRI) in refractory focal epilepsy patients. The authors investigate the effective reach of the hemodynamic changes related to icEEG IEDs, finding that these events can lead to associated BOLD changes across the whole brain. Furthermore, the authors investigate the correspondence between the BOLD and the topographic distributions of the IEDs, indicating that non-focal epileptiform discharges can generate a more accurate localization of the EZ. Cohen et al. investigate patients with epilepsy secondary to Polymicrogyria (PMG), a malformation of cortical development commonly associated with an intricate and widespread pattern of structural malformation (7). The results show that for this specific syndrome, the BOLD activations overlap the lesions in agreement with the scalp EEG detection in all studied cases. The choice for the hemodynamic response function (HRF) and its time delay relative to the target event is the subject of many EEG-fMRI studies (8, 9). Each epileptic syndrome is characterized by a constellation of factors that lead to specific epileptiform discharge propagation and hemodynamic behavior. The standard general linear model (GLM) with the canonical HRF provides robust results, but the model can benefit from a syndrome- or subject-specific response function modeling to improve the sensibility (10).

Labounek et al. performed a controlled experiment aiming to characterize data-driven EEG-fMRI fusion models in identifying task-related networks. They compared four EEG power models (two spectral and two spatial-spectral) as input on GLMs for individual EEG-fMRI fusion. The results indicate that the variable HRFs methodology significantly improves task-related BOLD responses throughout an entirely data-driven methodology.

The study of Ebrahimzadeh et al. proposes new modeling for the GLM variable of interest on EEG-fMRI analysis, finding promising results in terms of SOZ localization. The methodology consists of a data-driven EEG independent component selection using a previously defined subject-specific spike template. The regressor of interest is the select EEG component convolved with the canonical HRF.

Finally, Suarez et al. investigated the mechanisms that underlie negative BOLD responses (NBR) using the Windkessel (balloon) hemodynamic models. Based on four commonly discussed mechanisms, these findings indicate that an optimized GLM that considers the biophysical nature of the epileptic brain networks would improve the EEG-fMRI technique in precisely defining the SOZ. This study provides further insight into the meaning of NBR that is often seen in the EEG-fMRI maps of patients with epilepsy.

In conclusion, the articles in this Research Topic cover several aspects that are sensitive to the EEG-fMRI field, addressing each technique separately and the peculiar challenges of its combination. Studies have shown successful SOZ localization and indicate promising clinical applications for the method; however, there is still space for improvements regarding new methodological approaches and hardware development. We understand that the field can benefit from a standardization of acquisition and processing protocols among centers, aiming to increase reliability and reproducibility to favor a consolidated clinical application.

## Author Contributions

All authors listed have made a substantial, direct, and intellectual contribution to the work and approved it for publication.

## Conflict of Interest

The authors declare that the research was conducted in the absence of any commercial or financial relationships that could be construed as a potential conflict of interest.

## Publisher's Note

All claims expressed in this article are solely those of the authors and do not necessarily represent those of their affiliated organizations, or those of the publisher, the editors and the reviewers. Any product that may be evaluated in this article, or claim that may be made by its manufacturer, is not guaranteed or endorsed by the publisher.
